# ALDH-1-positive cells exhibited a radioresistant phenotype that was enhanced with hypoxia in cervical cancer

**DOI:** 10.1186/s12885-020-07337-8

**Published:** 2020-09-17

**Authors:** Tingting Yao, Xueling Weng, Yao Yao, Chunxian Huang, Jing Li, Yongpai Peng, Rongchun Lin, Zhongqiu Lin

**Affiliations:** 1 Department of Gynecological Oncology, Sun Yat-sen Memorial Hospital, Sun Yat-sen University, 107 Yan Jiang West Road, Guangzhou, 510120 People’s Republic of China; 2 Key Laboratory of malignant tumor gene regulation and target therapy of Guangdong Higher Education Institutes, Sun Yat-sen University, Guangzhou, China; 3 Guangdong Food and Drug Vocational College, Guangzhou, 510520 Guangdong China

**Keywords:** Cervical carcinoma, ALDH-1, Radioresistance, Hypoxia

## Abstract

**Background:**

We have previously found there was a small subpopulation of cells with cancer stem cell-like phenotype ALDH-1 in cervical cancer. Radiotherapy has been applied in most of the cervical cancer. However,the mechanisms underlying radioresistance still remained elusive. Our study is to explore whether ALDH+ cell promotes radioresistance by hypoxia.

**Methods:**

Cells were respectively cultured in hypoxia and normoxia environment and analyzed for marker stability, and cell cycle distribution. Results: Cell growth, apoptosis, cell cycle, sphere formation were affected by hypoxia. ALDH-1 and CHK2 were upregulated after hypoxia.

**Conclusions:**

Here we show that ALDH-1 positive cells contribute to cervical carcinoma radioresistance through preferential activation of the DNA damage checkpoint response and an increase in DNA repair capacity. The fraction of these cells is enriched after radiation in cervical carcinoma.

## Background

Cervical cancer is the second most frequent cancer in female worldwide, and radiotherapy (RT) has been considered as the key treatment modality for cervical carcinoma. More than 60% of cervical cancer patients have chosen radiotherapy as treatment [1]. Although improvement of control and survival has been shown after simultaneous chemo-radiation [2–4], impaired RT response is a major clinical problem in several solid tumor types including cervical carcinoma. As for advanced cervical cancer, nearly half of them got RT failure [5]. Therefore, new therapeutic approaches are needed to settle the radioresistance.

Most solid tumors have been identified with different oxygen areas [6]. Hypoxia is characterized by a hypoxic state, which is common in malignant tumors [7]. Hypoxia causes therapeutic resistance especially for radiotherapy. Hypoxia could generate reactive oxygen species and change the expression of proteins related to the repair of double stranded DNA and then dysregulating cell cycle checkpoint control leading to an abnormal DNA repair pathways [8–10]. These various effects related to hypoxia may help protect and maintain the cancer stem cell phenotype, thereby promoting tumor recurrence after treatment [11].

In our previous study, we found cervical cancer contains a small subpopulation of cells which may be associated with a cancer stem cell-like phenotype ALDH-1 [12]. In this study, we analyzed one mecanism of cervical cancer radioresistance, in order to improve prognosis.

## Methods

### Cell Cuture

The Hela and Siha cell lines were purchased from the Cell Bank of the Chinese Academy of Sciences (Shanghai, China) and cultured in DMEM-high glucose medium (GIBCO) supplemented with 10% newborn calf serum (GIBCO).

### Establishment of radioresistant cell line

X-ray resistant sublines were produced by continuous sublethal radiation for 6 months, and 2Gy radiation was repeated 35–38 times with a total dose of 70–76 Gy. The parental cell lines were treated under the same conditions without ionizing irradiation, which have been proved in our previous studies [13, 14]. Pictures were captured utilizing the microscope(cellSens Standard 1.18, IX71, OLYMPUS. Objective lenses: 20x).

### Hypoxia treatment

Normoxia condition was in a humidified atmosphere of 20% O_2_, 5% CO_2_ and 94% N_2_ at 37 °C. Hypoxic condition was established using an airtight anaerobic incubator containing 1% O_2_, 5% CO_2_, and 94% N_2_ to culture cells for 4 hs each day at 37 °C before experiment [15].

### Cell proliferation assays

The cell survival was assessed using the 3-(4, 5-dimethylthiazol-2-yl)-5-(3- carboxymethoxyphenyl)- 2(4-sulfophenyl)-2H-tetrazolium (MTS) assay (Sigma). For the MTS assay (Promega), 1 × 10^4^ cells per well from each cell line were seeded triplicately in a 96-well plate. At 1st d, 2nd d,3rd d, 4th d and 5th day, 20 μL of MTS was added to each well and incubated for 1 h at 37 °C; the results were analyzed by a plate reader at 490 nm. The sample data were normalized to the background readings of media only [16].

### Apoptosis analysis

The percentage of apoptosis in radioresistant cells treated with different oxygen concentration was detected by flow cytometry. The cell pellets were resuspended in AnnexinV- binding buffer (Roche Molecular Biochemicals) and incubated for 15 min at room temperature. PI was stained afterward.

### Cell cycle analysis

After different oxygen treatment, the radioresistant cells were harvested and fixed in 70% ice cold ethanol and followed by RNase A treatment, stained with 50 lg/mL of propidium iodide for the DNA content analysis by flow cytometry on a FACS Calibur system (EPICS ALTRA, Beckman Coulter, Fullerton, CA). The data were processed by FlowJo FACS analysis software (Tree Star, Ashland, OR) [16].

### Sphere formation assay

Single cell suspensions of cells were seeded at 1000 cells/well on ultra low adherent wells of 6-well plate (Corning, Lowell, MA) in sphere formation medium. Medium was refreshed every 3–4 days. At day 10, spheres larger than 50 μm were counted under lightmicroscope. Sphere-forming efficiency (SFE) was calculated as the number of spheres formed divided by the original number of single cells seeded and expressed as a percentage. All experiments were done in triplicate.

### Immunoblot analysis

Phospho-Chk2 (Thr68) (Cell signaling technology) was visualized with horseradish peroxidase–conjugated secondary antibodies and chemiluminescence was purchased from Amersham, Pittsburgh, PA. β-actin was chosen as control.

### Flow cytometry analysis for ALDH-1

Experimental tubes were added with 2 μl activated Aldefluor reagent with BAAA, whereas control tubes were added with 2 μl of ALDH reaction inhibitors. Experimental tubes were then added 400 μl of the cell suspension with the adjusted concentration, 200 μl of which was immediately transferred after mixing to the corresponding control tubes. Experimental and control tubes were incubated for 0.5-1 h at 37 °C in the dark. They were centrifuged for 5 min at 1000 rpm. Then resuspend the cells with Aldefluor Assay Buffer and analyzed with a flow cytometer (Beckman-coulter MoFlo XDP).

### Tumor xenografts

According to the Guide for the Care and Use of Laboratory Animals (NIH publication nos. 80–23, revised 1996), 4–6-week-old female nude mice from SunYat-Sen University Laboratory Animal Center (Guangzhou, China) were cared for animal experiments and approved by the Animal Research Committee of Sun Yat-Sen University.Hela-RR cells (1 × 10^6^) treated with normoxia or hypoxiabefore were suspended in 200 μl PBS and then injected subcutaneously into either side of the posterior flank of the same female nude mouse. Twenty –four nude mice were used in our experiment. Tumor growth was detected every 3 days. All mice were humanely euthanized with an intraperitoneal injection of pentobarbital sodium at the end of the experiment.

### Statistical analysis

Data were presented as the mean ± standard deviation (SD). Statistical analyses were performed with SPSS 20.0 software using two-tailed Student’s t-tests, Chi-square tests and Log-rank tests. The difference was considered statistically significant at < 0.05. All experiments were repeated independently in triplicate.

## Results

### Hypoxia drove morphological changes of cervical radioresistant cells

**Fig. 1:**
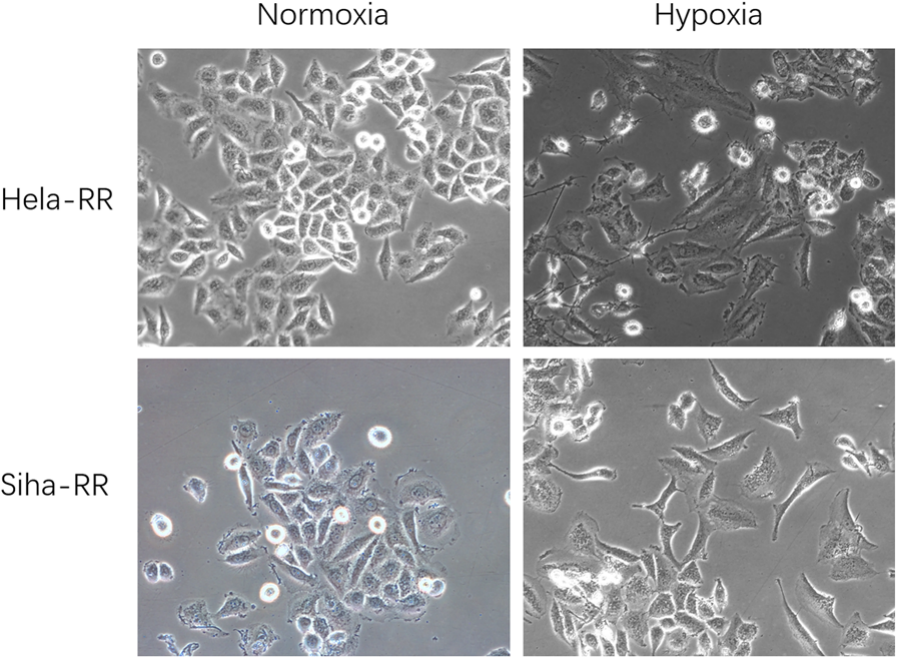
Cell morphology in different oxygen condition. **a** Normoxia **b** Hypoxia

We studied the morphology of cells under a microscope. When exposed to hypoxic conditions, cells exhibited typical morphological changes, appearing flat, spindle- shaped and fibroblast-like, lack of cytoplasmic protrusions and intercellular connections. Cells under normoxic control showed strong cell connections (Fig. 1).

### Hypoxia stimulated the growth of Hela-RR and Siha-RR cell lines

**Fig. 2:**
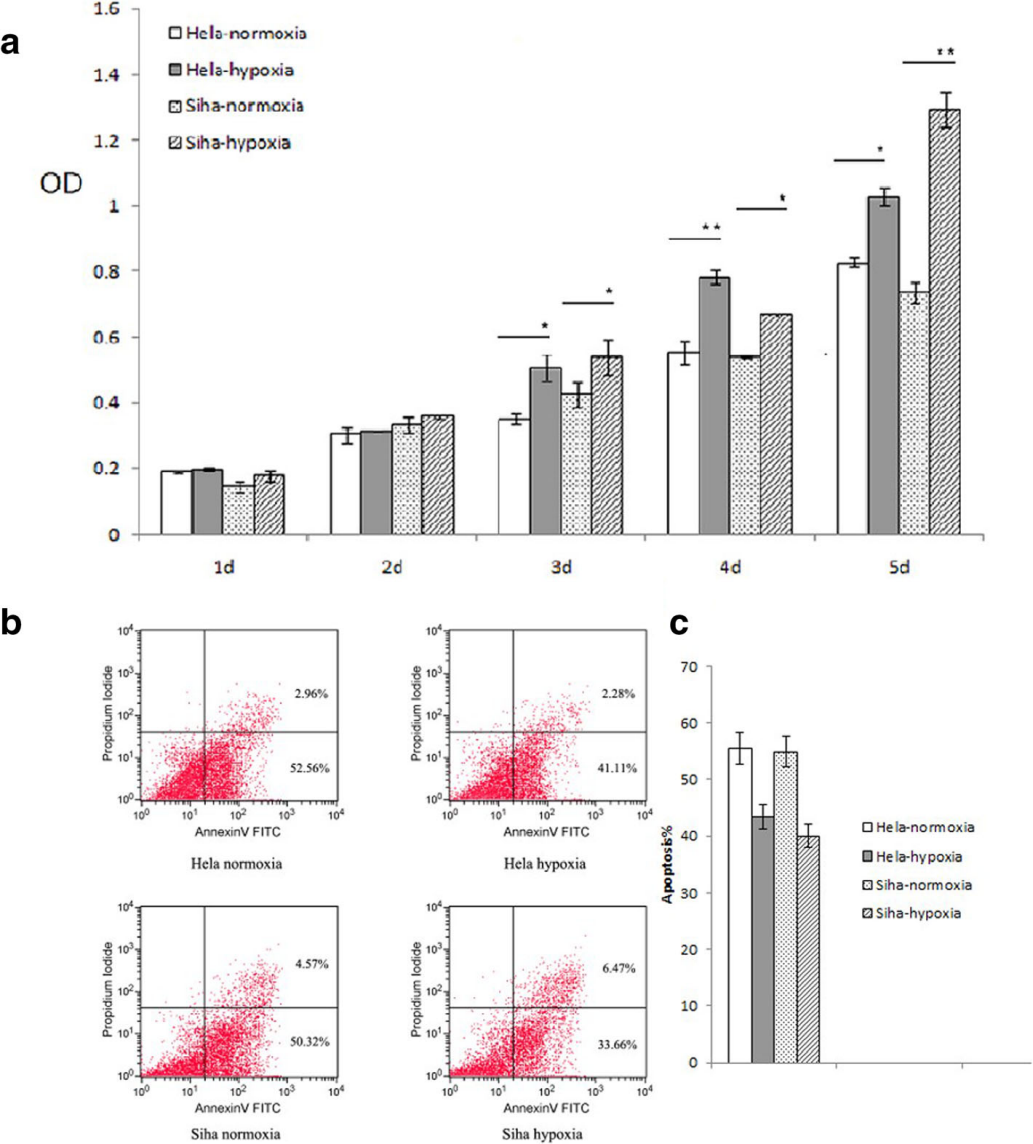
Hypoxia stimulated the growth and decreased cell apoptosis. **a** Viability was analyzed with MTS assay. Results were from one representative experiment repeated in triplicate and presented as mean ± SEM with **P < 0.01 and *P < 0.05, respectively. **b** The apoptosis at 72 h after Hela-RR and Siha-RR cells treated with hypoxia or normoxia. **c** Results were from one representative experiment repeated in triplicate and presented as mean ± SEM with **P < 0.01 and *P < 0.05, respectively

MTS assays were used to examine the growth of cells (Fig. 2a). The result showed that the proliferation of Hela-RR and Siha-RR treated with hypoxia previously were faster than those of under normoxia after 3 days(*P* < 0.05) .

### Hypoxia decreased cell apoptosis

As viability of cells was decreased by radiation, we assessed the apoptotic populations by Annexin V/PE staining. By contrast, compared to the findings under hypoxia, the apoptotic population was slightly lower after normoxia treatment (*P* < 0.05) (Fig. 2b & c).

### Hypoxia modulated cell cycle progression

**Fig. 3:**
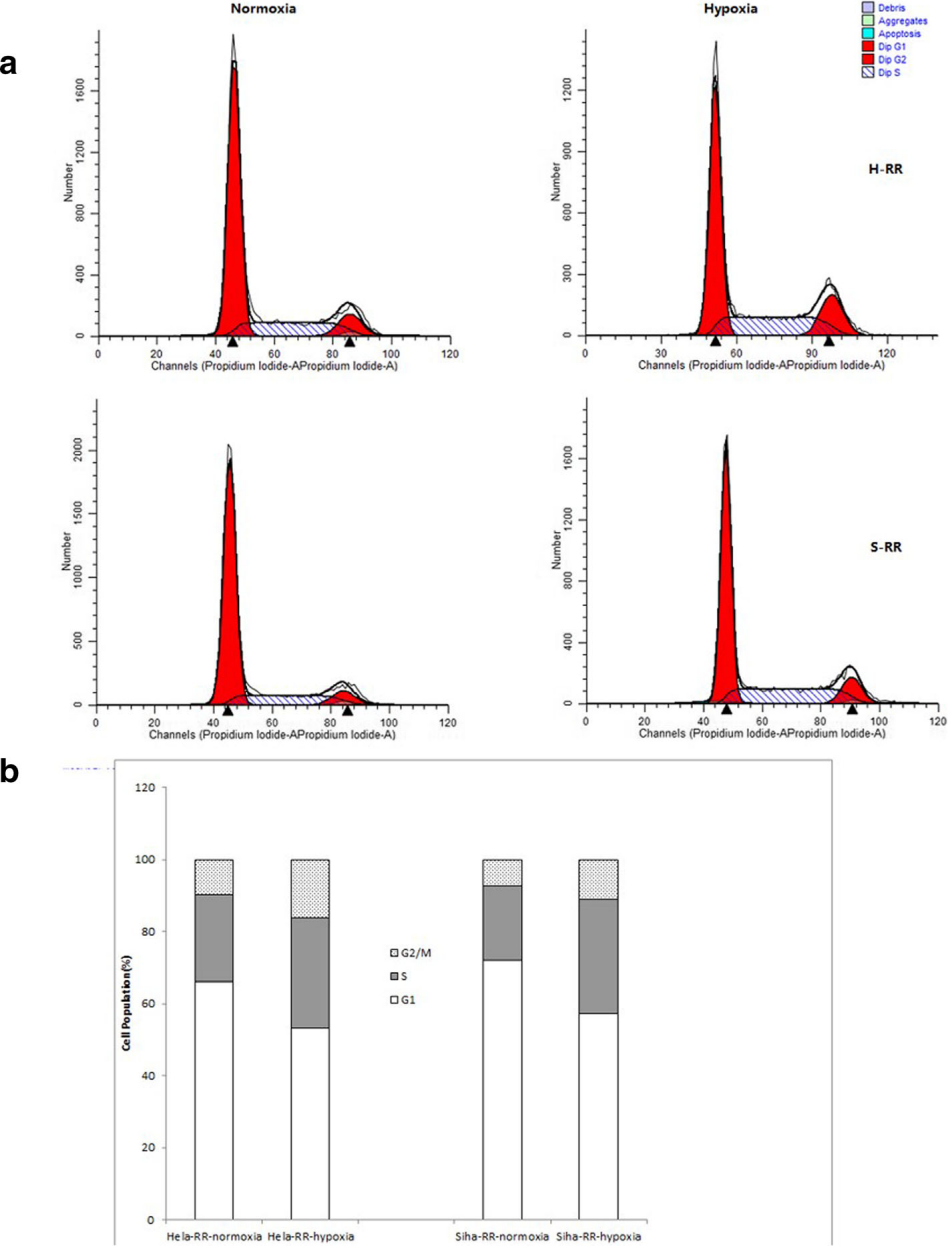
The cell cycle detected on 3rd day with different oxygen concentration treatment. **P < 0.01 and *P < 0.05, respectively

The cell cycle changes were investigated after normoxia or hypoxia treatment to identify the possible action mechanism. The results for hypoxia treatment showed accumulation of S phase (*P* < 0.05) (Fig. 3a & b).

### Hypoxia enhanced sphere formation in radioresistant cells

**Fig. 4:**
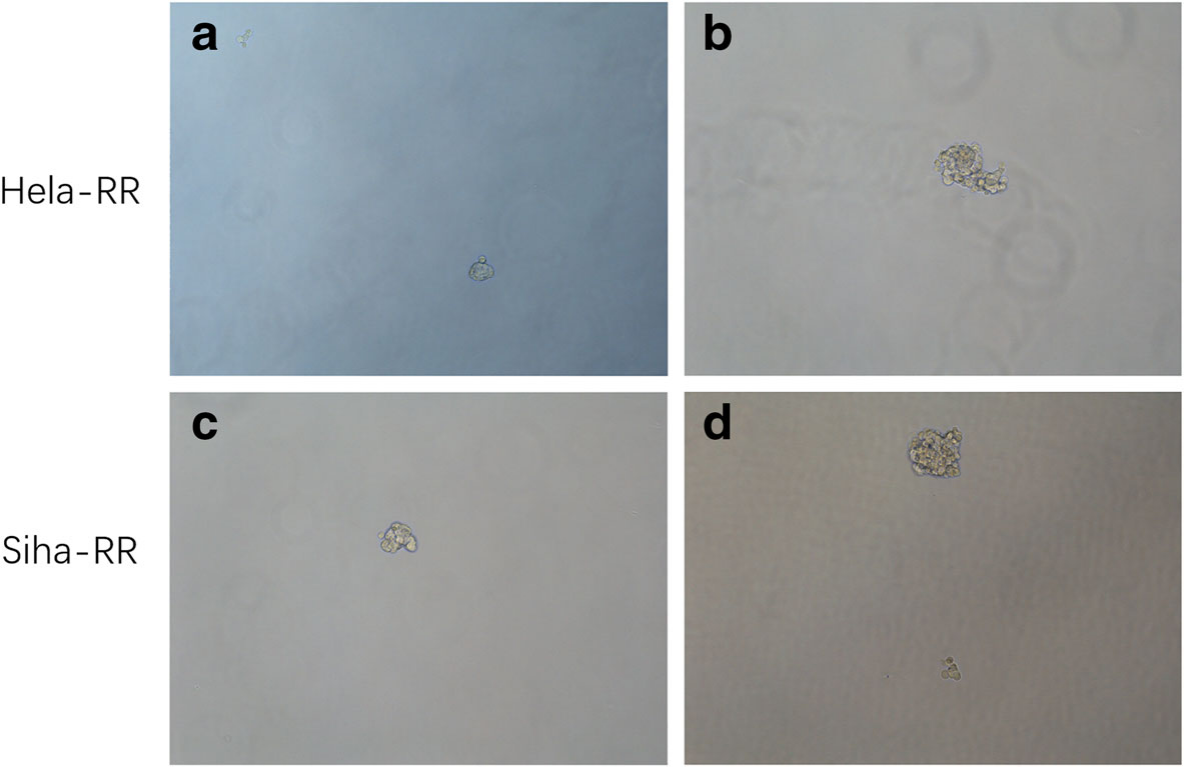
Sphere formation of ability after cells treated with normoxia (**a** & **c**) or hypoxia (**b** & **d**)

Non-adherent spheroids formation has been widely used to assess cancer stem cell characteristics. It was recently demonstrated that cervical CSCs could form tumor spheres [17–21]. Therefore, we investigated the sphere-forming activity. Compared with normoxia cells, resistant cells had significantly higher sphere-formation efficiency under hypoxia condition (*P* < 0.05) (Fig. 4).

### Hypoxia promoted ALDH-1 expression in radioresistant cells

**Fig. 5:**
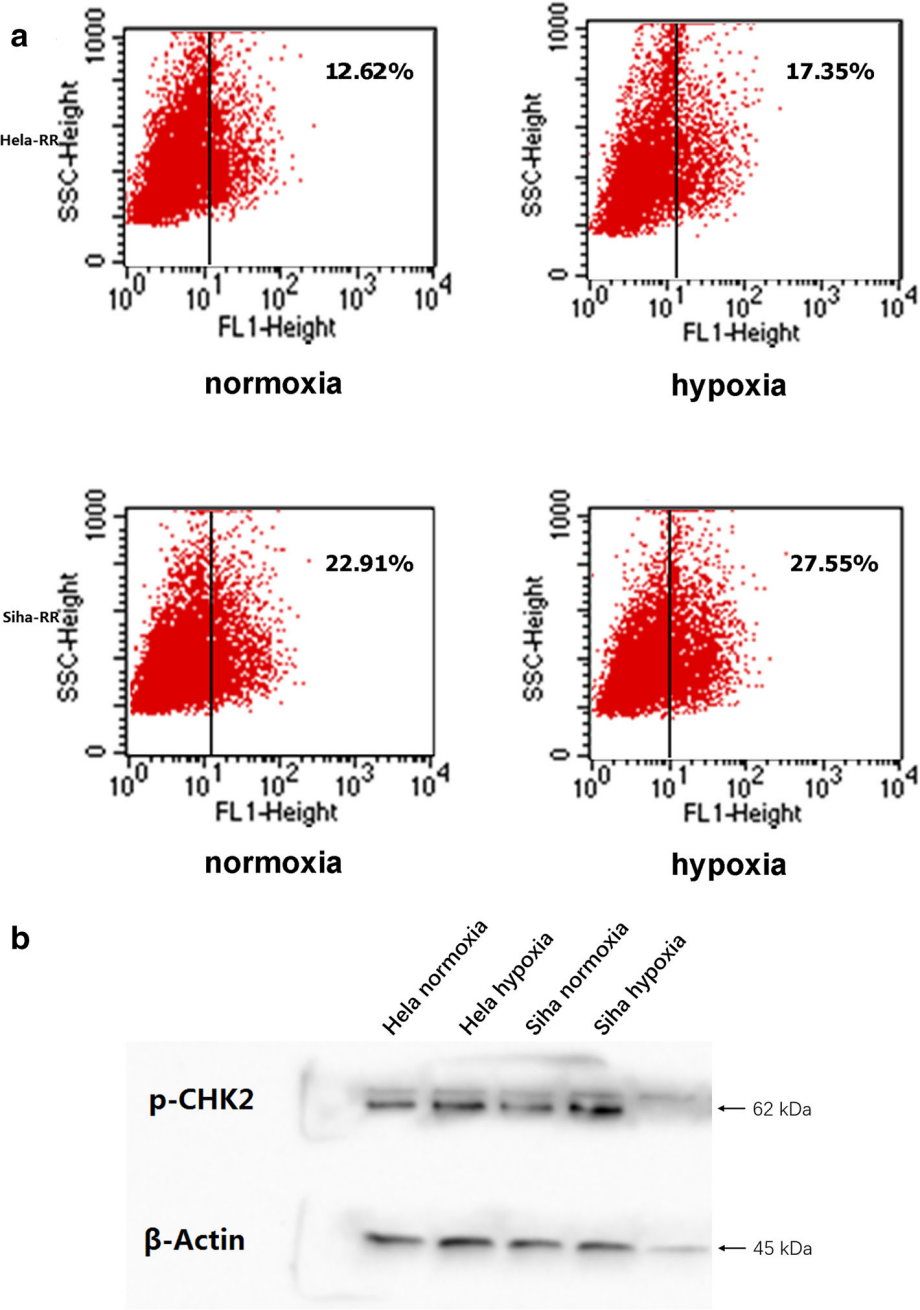
**a** Identification of a small ALDH1^+^ population by FACS analysis using the Aldefluor assay DEAB, an inhibitor of ALDH, was used for negative control. (left) The percentage of ALDH1^+^ under hypoxia. (right) The percentage of ALDH1^+^ of Hela under normoxia. **b** The expression of p-Chk2 was upregulated under hypoxia

It has been reported that ALDH-1 might be one of markers of cancer stem cell. We then determined whether ALDH-1 was indeed upregulated in hypoxic radioresistant cells. Our results showed resistant cells received hypoxia treatment expressed more ALDH-1 than normoxia cells (Fig. 5a).

### Expression of DNA damage checkpoint CHK2 was upregulated in hypoxia

Checkpoints are initiated to ensure DNA replication and chromosome segregation of the cell cycle. To check whether the marked induction of S phase in hypoxia by cell cycle regulator, we examined the expression of Chk2 using western blotting. We did observe an accumulation of phosphorylated Chk2 (Thr 68) (Fig. 5b).

### In vivo

**Fig. 6:**
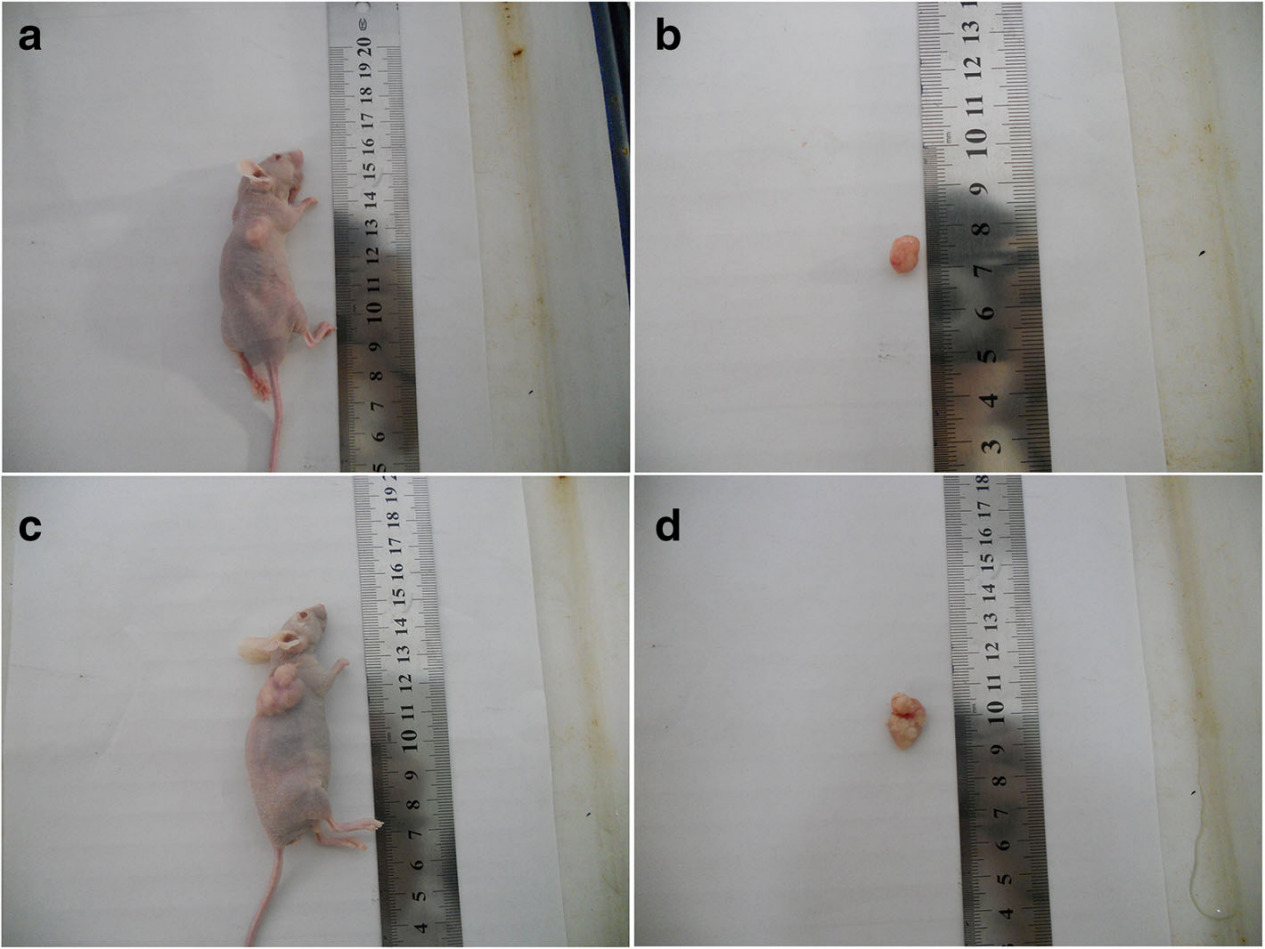
Effect of hypoxia condition on tumor formation in a nude mouse xenograft model. **a b** Hela-RR under normoxia **c d** Hela-RR under hypoxia

To further examine the effect of hypoxia on the in vivo growth of cervical carcinoma, Hela-RR cells were independently injected subcutaneously into either anterior flank of the same nude mouse. Compared to the cells grown in normoxia, the frequency of tumor formation was not significantly increased after hypoxia,while the volume of the tumor was larger (Fig. 6).

## Discussion

In 2018,there were 569,847 diagnosed as cervical cancer in the world, and 311,365 died of cervical cancer [22]. The effect of radiotherapy is equal to radical surgery in early stage cervical cancer,while for advanced cervical cancer,it is advocated a combination of irradiation and cisplatin-based chemotherapy [23]. So radiotherapy is very important to cervical cancer. However some of them developed into radioresistance and the mechanism of acquired radioresistance is still obscure. The cells induce an adaptive response to chronic exposure to IR, resulting in increased tolerance to subsequent cytotoxicity of IR [24]. In our previous study, a radioresistant subline/R was obtained by exposing the cell line with fractional X-rays. This resulted in a statistically significant decrease in the radiosensitivity of the exposed subline [7, 8].

Cancer stem cells are a sub-population of cells which could self-renew and maintain tumorgenity. At present, there are several studies indicating that CSCs are more radioresistant than other cancer cells. Bao and coworkers [25] reported that the fraction of tumor cell fractions expressing CD133 (Prominin-1) (a marker of neural stem cells and brain cancer stem cells) were enriched after radiation in gliomas. They proposed that these cells represented a population of cells that conferred radioresistance to gliomas and might be a source of tumor recurrence after radiation. Phillips et al. [26] reported that cancer-initiating cells were more resistant to radiation than monolayer-cultured cells and fractionated doses of irradiation increased the cancer-initiating cells percentage in the non-adherent MCF-7 monolayer cell cultures. They considered that breast cancer-initiating cells were a relatively radioresistant subpopulation. These researches indicated that radioresistant sub-lines were rich of CSCs. We have successfully obtained radioresistant cervical cancer cell sub-lines by repeated X-ray radiation. And our previous study showed cervical carcinoma contained a small subpopulation of cells that may relate to a cancer stem cell-like phenotype ALDH-1 [1, 27, 28]. Then we found that the rate of ALDH-1 increased distinctly under hypoxia. It pointed out that ALDH-1^+^ cells were more radioresistant than ALDH-1^−^ cells and inferred ALDH-1 might be used as one of the markers of cervical cancer stem cell.

Among various internal and external factors, hypoxia has received considerable attention in recent years because they have been reported to be associated with poor prognosis, local tumor recurrence and distant tumor metastasis after radiation therapy [29–34]. For cervical cancer, hypoxia is associated with poor prognosis and resistance to radiation therapy [35–37]. It has also demonstrated that local control of cervical cancer reoxygenation patients is significantly better. Based on these, radiation-induced reoxygenation is intended to make radioactive tumor cells during radiotherapy more radiosensitive.

Our data showed that hypoxic cells had a survival advantage compared to oxygenated cells. This survival advantage was associated with induction of S phase and decreased apoptosis. Hypoxia has been proved suppressing DNA repair through homologous recombination (HR) and inducing cell cycle arrest in radiation-sensitive G1 phase [38–40]. However, the molecular mechanism behind the transition of cell cycle under hypoxic conditions is still unknown.

Activating checkpoints in response to DNA damage often cause cell cycle arrest. Checkpoints are initiated to ensure the orderly and timely completion of DNA replication and chromosome segregation. It has been reported that DNA damage checkpoint responses play important roles in cellular radiosensitivity. Overexpression of cyclin D1 is associated with fractional radiation-induced acquired radioresistance in HeLa cells. Inhibition of cyclin D1 by using small interfering RNA (siRNA) reduced radioresistance [41] .Chk2 is activated at the DNA double-strand break. This mechanism requires phosphorylation on threonine 68 and is dispersed throughout the nucleus of the irradiated cells. Our accumulation of phosphorylated Chk2 (Thr 68) after radiation treatment was in accordance with previous studies.

In order to test tumorigenicity in vivo, we injected the same number of spheroid cells of hypoxia and normoxia into nude mice. Although there was no difference between these two groups, the volumes of the hypoxia group were much larger than the other group. It suggested that these spherical cells derived from cervical cancer under hypoxia had more capacity of proliferation.

## Conclusion

Our data showed that hypoxia exposure was important in the development of radioresistance and suggested that targeting hypoxia after radiation coould benefit patients with invasive hypoxic cervical cancer.

## Supplementary information

**Additional file 1.**

**Additional file 2.**

## Data Availability

The datasets generated during and/or analysed during the current study are available from the corresponding author on reasonable request.

## Abbreviations

ALDH-1: Aldehyde dehydrogenase 1
RT: Radiotherapy
RR: Radioresistance
CSC: Cancer stem cell
IR: Ionizing radiation
DNA: Deoxyribonucleic acid
RNA: Ribonucleic Acid
