# Supplementary material for: ALDH-1-positive cells exhibited a radioresistant phenotype that was enhanced with hypoxia in cervical cancer
Source: BMC Cancer. 2020 Sep 17;20:891. doi: 10.1186/s12885-020-07337-8 (PMC7499852; doi:10.1186/s12885-020-07337-8)
Supplement: Supplementary file 1 — Additional file 1. [file 12885_2020_7337_MOESM1_ESM.pdf]

Report of Human Cell Line Authentication

Delivery Date: Aug 8th, 2017

Analysis Date: Aug 16th, 2017

Ⅰ. Sample

Sample Name: ‘JD1771’, labeled as ‘A’, and was received on Aug 8th, 2017

Ⅱ. Method and Procedure

1. PCR is amplified with STR Multi-amplification Kit (PowerPlex™16HS System);
2. PCR products are assayed with 3100 DNA Analyzer (Applied Biosystems®).
3. Amplification of gene COX1 and electrophoresis are employed to survey the species of the sample.

Ⅲ. Results

1. The STR profiles of the cell line sample are in the attached table and figure.
2. The search result in ATCC and DSMZ databases.
3. The electrophoresis figure of gene COX1.

A: ①No loci has tri-alleles or tetra-alleles. Contamination of other human cell line is not found (Figure 1 & Table 1). ②100% matched cell lines are found in ATCC and DSMZ data banks. And the cell line named as “HELA” *et al*. (Figure 2 & Figure 3) ③The sample is a human cell line. Contamination of other species cells are not found in the sample (Figure 4).

Operator: Xiaohua Mo

Auditor: Xuanyi Liang

Guangzhou Cellcook Biotech Co., Ltd

GUANGZHOU CELLCOOK BIOTECH CO.,LTD
16 AUG 2017
CELL STR PROFILING PASS

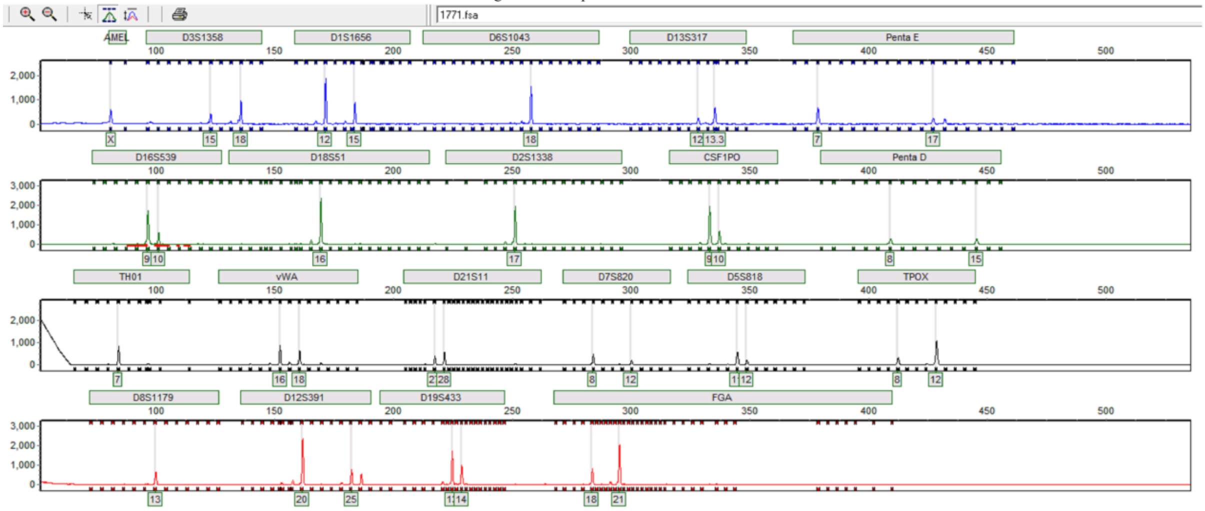

Figure 1. STR profiles of A cell line

Table 1. STR profiles of A cell line

	Allele1	Allele2
AMEL	X	
D3S1358	15	18
D1S1656	12	15
D6S1043	18	
D13S317	12	13.3
Penta E	7	17
D16S539	9	10
D18S51	16	
D2S1338	17	
CSF1PO	9	10
Penta D	8	15
TH01	7	
vWA	16	18
D21S11	27	28
D7S820	8	12
D5S818	11	12
TPOX	8	12
D8S1179	13	
D12S391	20	25
D19S433	13	14
FGA	18	21

Figure 2. Search result in ATCC database

SEARCH THE STR DATABASE

As part of our continuing efforts to characterize and authenticate the cell lines in the Cell Biology collection, ATCC has developed a comprehensive database of short tandem repeat (STR) DNA profiles for all of our human cell lines. View our brief tutorial before starting.

1. STR Profiling Analysis
2. Matching Algorithm
3. Interrogating the Database

Showing 1 - 20 Of 20

PageSize: 100

Add to Cart	%Match	ATCC® Number	Designation	D5S818	D13S317	D7S820	D16S539	vWA	TH01	AMEL	TPOX	CSF1PO
	100.0	CCL-2	HeLaCervical AdenocarcinomaHuman	11,12	12,13.3	8,12	9,10	16,18	7	X	8,12	9,10
	100.0	CCL-5	L132Cervical carcinomaHuman	11,12	12,13.3	8,12	9,10	16,18	7	X	8,12	9,10
	100.0	CCL-6	Intestine 407HeLa ContaminantHuman	11,12	12,13.3	8,12	9,10	16,18	7	X	8,12	9,10
	100.0	CCL-13	Chang LiverHeLa ContaminantHuman	12	12,13.3	8,12	9,10	16,18	7	X	8,12	10
	100.0	CCL-17	KBHeLa ContaminantHuman	11,12	12,13.3	8,12	9,10	16,18	7	X	8,12	9,10
	100.0	CCL-21	AV-3AmnionHuman	11,12	13.3	12	9,10	16,18	7	X	8,12	9,10

Figure 3. Search result in DSMZ database

Result of STR matching analysis by your data.

- DSMZ Profile Database -

A graphical presentation is shown at the bottom of this page.

EV	Cell No.	Cell name	Locus names									Figures
			D5S818	D13S317	D7S820	D16S539	VWA	TH01	AM	TPOX	CSF1PO	
	Query (Your Cell)		11,12	12,13.3	8,12	9,10	16,18	7,7	X,X	8,12	9,10	
1.00(36/36)	57	HELA	11,12	12,13.3	8,12	9,10	16,18	7,7	X,X	8,12	9,10	-
1.00(36/36)	57	HELA	11,12	12,13.3	8,12	9,10	16,18	7,7	X,X	8,12	9,10	-
1.00(36/36)	116	GIRARDI HEART C2	11,12	12,13.3	8,12	9,10	16,18	7,7	X,X	8,12	9,10	-
1.00(36/36)	121	GIRARDI HEART C7	11,12	12,13.3	8,12	9,10	16,18	7,7	X,X	8,12	9,10	-
1.00(36/36)	136	KB	11,12	12,13.3	8,12	9,10	16,18	7,7	X,X	8,12	9,10	-
1.00(36/36)	149	KB-V1	11,12	12,13.3	8,12	9,10	16,18	7,7	X,X	8,12	9,10	-
1.00(36/36)	158	KB-3-1	11,12	12,13.3	8,12	9,10	16,18	7,7	X,X	8,12	9,10	-
1.00(36/36)	161	HELA-S3	11,12	12,13.3	8,12	9,10	16,18	7,7	X,X	8,12	9,10	-
1.00(36/36)	CCL-17	KB	11,12	12,13.3	8,12	9,10	16,18	7,7	X,X	8,12	9,10	-
1.00(36/36)	CCL-2	HeLa	11,12	12,13.3	8,12	9,10	16,18	7,7	X,X	8,12	9,10	-
1.00(36/36)	CCL-2.1	HeLa 229	11,12	12,13.3	8,12	9,10	16,18	7,7	X,X	8,12	9,10	-

Figure 4. Authentication of the species of the sample

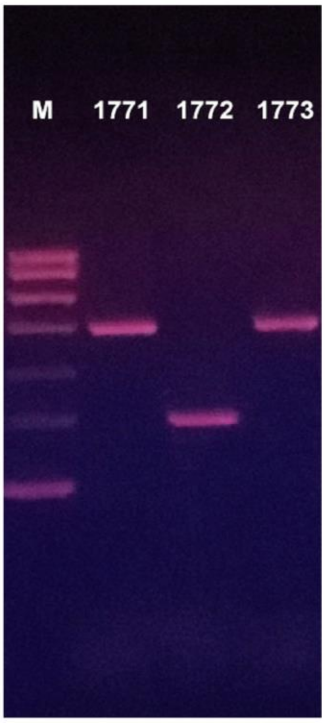

M: Marker. As the size of 700, 600, 500, 400, 300, 200 and 100bp from up to down.

Nine species are checked, as follow: *Homo sapiens* 391bp, *Cricetulus griseus*315bp, *Macaca mulatta*287bp, *Cercopithecus aethiops*222bp, *Rattus norvegicus*196bp, *Canis familiaris*172bp, *Mus musculus*150bp, *Bos Taurus*102bp, IC 70bp

JD1771: The sample. The band size is 391bp which matches the size of human.
