# Supplementary figures and images for: ALDH-1-positive cells exhibited a radioresistant phenotype that was enhanced with hypoxia in cervical cancer
Source: BMC Cancer. 2020 Sep 17;20:891. doi: 10.1186/s12885-020-07337-8 (PMC7499852; doi:10.1186/s12885-020-07337-8)

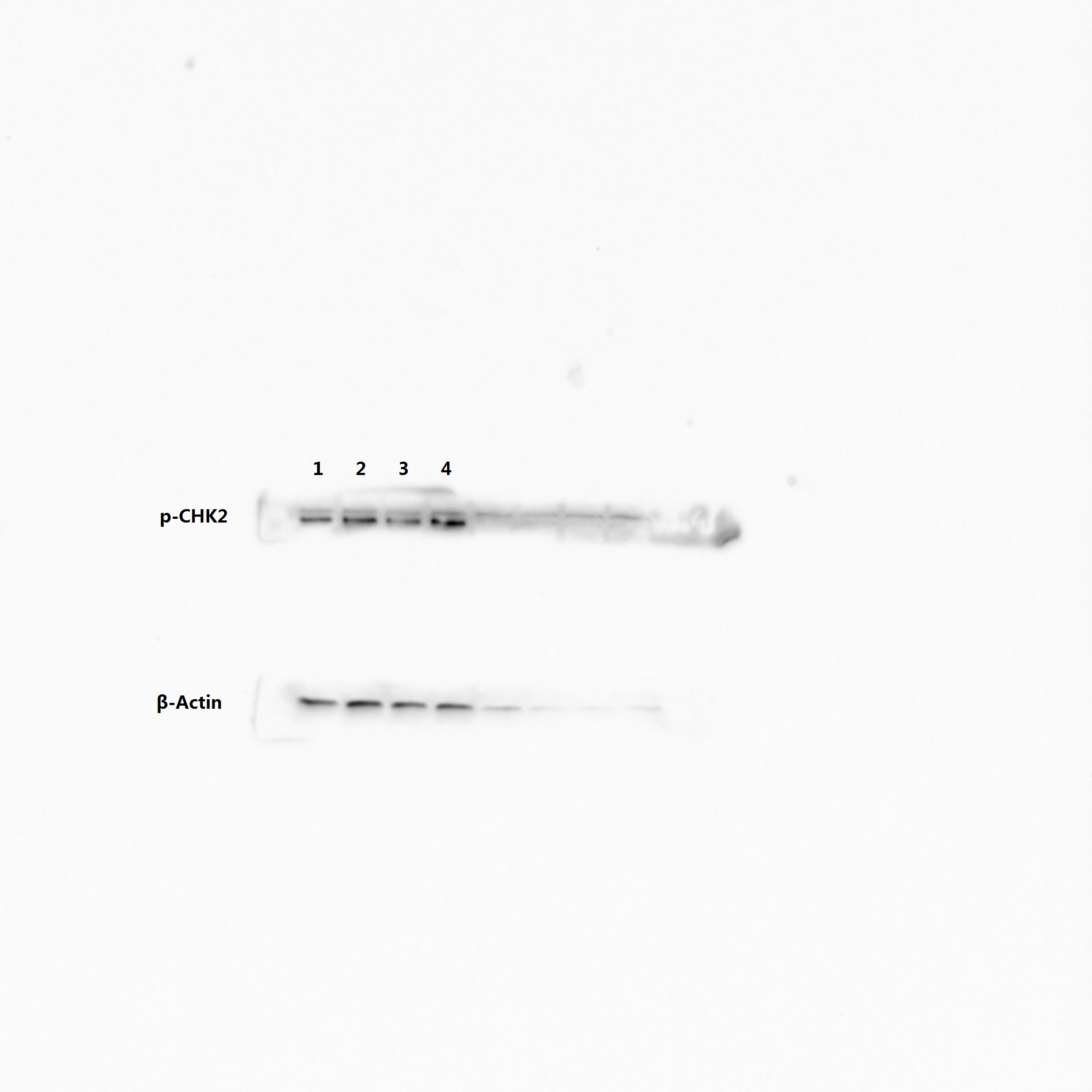

Supplement: Supplementary file 2 — Additional file 2. [file 12885_2020_7337_MOESM2_ESM.tif]
